# Supplementary material for: Identification of photoperiod-induced specific miRNAs in the adrenal glands of Sunite sheep (Ovis aries)
Source: Front Vet Sci. 2022 Jul 22;9:888207. doi: 10.3389/fvets.2022.888207 (PMC9354845; doi:10.3389/fvets.2022.888207)
Supplement: Supplementary file 1 [file Data_Sheet_1.ZIP › Supplementary file/miRDeep2 file.zip!/miRDeep2 file/Know_Structure/SP42A3/oar-mir-541.pdf]

miRBase precursor : oar-mir-541
Total read count : 149
oar-miR-541-5p read count : 143
oar-miR-541-3p read count : 5
remaining reads : 1

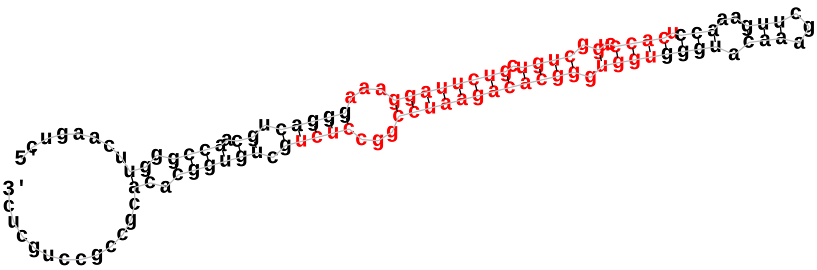

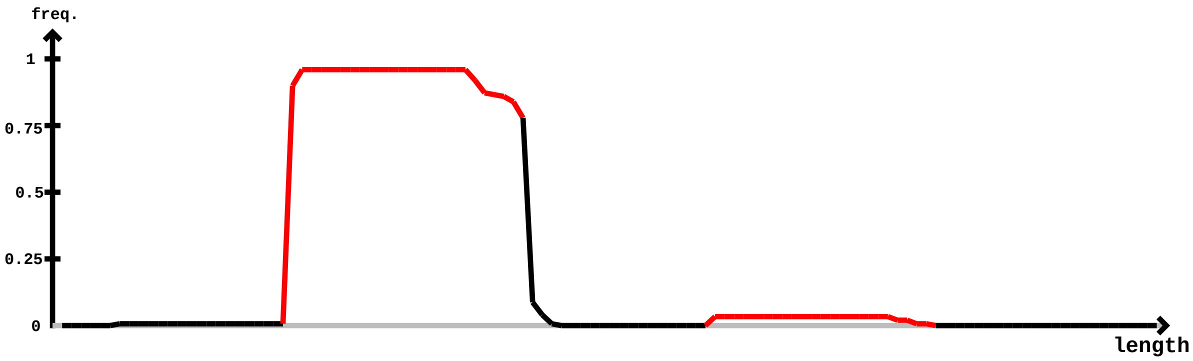


```
                                                                   oar-miR-541-3p
                      oar-miR-541-5p
5'- cugaacuugggccaacgucagggaaaggauucugcugucggucccacuccaaaguucgaaacauggguggugggcacagaauccggccucugcuguggcacacgccgccugcuc  -3'   exp
    ........((((..(((.(((((...((((((((.((((.(..((((.(((..(((...))).)))))))).))))))))))))...))))).)))(((....)))))))....   reads   mm   sample
    .....cuugggccaacgucaggg...........................................................................................   1       0    seq
    .......................aaaggauucugcugucggu........................................................................   6       0    seq
    .......................aaaggauucugcugucgguc.......................................................................   7       0    seq
    .......................aaaggauucugcugucggucc......................................................................   1       0    seq
    .......................aaaggauucugcugucgguccc.....................................................................   1       0    seq
    .......................aaaggauucugcugucggucccа....................................................................   3       0    seq
    .......................aaaggauucugcugucggucccac...................................................................   9       0    seq
    .......................aaaggauucugcugucggucccacu..................................................................   97      0    seq
    .......................aaaggauucugcugucggucccacuc.................................................................   7       0    seq
    .......................aaaggauucugcugucggucccacucc................................................................   2       0    seq
    .......................aaaggauucugcugucggucccacucca...............................................................   1       0    seq
    ........................aaggauucugcugucggucccacu..................................................................   6       0    seq
    ........................aaggauucugcugucggucccacucc................................................................   3       0    seq
    ...................................................................uggugggcacagaauccgg............................   2       0    seq
    ...................................................................uggugggcacagaauccggcc..........................   2       0    seq
    ...................................................................uggugggcacagaauccggccuc........................   1       0    seq
```
